# Durability Assessment of Cement Mortars with Recycled Ceramic Powders

**DOI:** 10.3390/ma18184420

**Published:** 2025-09-22

**Authors:** Anna Tokareva, Danièle Waldmann

**Affiliations:** 1 Department of Engineering, Faculty of Science, Technology and Medicine, University of Luxembourg, L-4365 Esch-sur-Alzette, Luxembourg; 2 Institute for Solid Structures, Technical University of Darmstadt, 64287 Darmstadt, Germany

**Keywords:** durability, sustainability, ceramic waste, limestone calcined clay, freeze–thaw, fire resistance, sulphuric acid

## Abstract

Although substantial knowledge exists regarding the use of ceramic powders as pozzolanic supplementary cementitious materials, a notable gap remains in the literature concerning the durability properties of cement with ceramics. This research aims to address this gap by evaluating the effects of ceramic powders on mortar durability, specifically focusing on resistance to freeze–thaw, high temperatures, and 1% sulphuric acid. The study also investigates the use of recycled ceramic demolition waste as a replacement for calcined clay in limestone calcined clay (LC3) formulations. This research demonstrates the potential of using ceramic waste to enhance mortar durability. The results show significant improvements in freeze–thaw resistance, with strength losses of 1.91% to 2.61% for modified mortars, compared to 6.31% for the reference mortar. Fire resistance also improves, with strength gains of up to 13.9% at 200 °C for LC3 mortars with ceramic powder. At 500 °C, strength losses ranged from 2.8% to 31.9%, with ceramic-containing mortars showing better performance than the reference. At 900 °C, substantial strength losses occurred across all mixes (72.0% to 90.0%), with mortars containing ultrafine ceramic powder showing the best resistance. Resistance to 1% sulphuric acid is enhanced, with strength losses decreasing from 9.37% in the reference mortar to 1.38% in LC3 mortar with ceramic powder.

## 1. Introduction

### 1.1. Literature Review

The utilisation of binders incorporating ceramic powder as a pozzolanic component has a long historical precedent, with evidence tracing back to ancient civilisations. In Ancient Rome, lime-based mortars mixed with ground ceramic materials, particularly brick dust, were extensively employed in construction. This practice was especially widespread in regions where natural pozzolanic materials, such as volcanic ash, were scarce or unavailable [1,2].

Numerous studies confirm the pozzolanic properties of ceramic powders and support their suitability as supplementary cementitious materials (SCMs) for the production of CO_2_-reduced cements. For instance, Partarch et al. [3] measured the amount of lime fixed by cement pastes containing 25 wt.% ceramic bricks, tiles, and sanitary ware, and confirmed the superior pozzolanic activity of bricks compared to other ceramic materials. Silveira et al. [4] confirmed the pozzolanic nature of waste sanitary ware using isothermal calorimetry, thermogravimetric analysis, electrical conductivity, and strength activity index. Kannan et al. [5] applied the Frattini test to cements containing 20% and 40% waste ceramics, demonstrating their pozzolanic reactivity at both 28 and 90 days. The study by Gonçalves et al. [6] reported lower strength in mortars containing brick powder compared to those with metakaolin at the same packing density, leading to the conclusion that brick powder has lower pozzolanic activity. Although the pozzolanic reactivity of ceramic powders is generally lower than that of conventional calcined clays such as metakaolin, it remains comparable to that of fly ash, which is widely used as a pozzolanic SCM in blended cements. Thus, Pang et al. [7], after testing the pozzolanic properties of ceramic polishing powder, fly ash, and blast furnace slag using the Rapid, Reliable, and Relevant (R3) method in accordance with ASTM C1897-20 [8], observed a similarity in pozzolanic activity between ceramics and fly ash.

Experimental investigations have demonstrated that the partial replacement of ordinary Portland cement (OPC) with finely ground ceramic waste enables the development of more sustainable binders with reduced environmental impact. Most research indicates that the optimal replacement level is in the range of 20–25% by mass. At this dosage, the compressive strength of mortars and concretes incorporating ceramic powders tends to match or surpass that of conventional OPC-based materials, though this improvement is typically observed only at later curing ages, beyond 28 days. Thus, Ebrahimi et al. [9] investigated the strength development of mortars with ceramic waste powders at 28, 56, and 90 days, and observed an increase in strength compared to OPC when the cement replacement rate was up to 20%. Faldessai et al. [10] found that the maximum 28-day compressive strength of concrete containing ceramic waste was achieved at a replacement rate of 25%, resulting in a 14.89% reduction in production cost compared to ordinary concrete. El-Dieb and Kanaan [11] proposed an optimal substitution rate of 10–20% for the best balance between strength and workability, but noted that higher ceramic powder content is required to improve durability. However, Pang et al. [7] concluded that the inclusion of more than 25% ceramic powder increases autogenous shrinkage, which may negatively affect durability.

Microstructural analyses have shown that the inclusion of ceramic powders enhances the density and compactness of the cementitious matrix. Silveira et al. [4], Kannan et al. [5], and Tremiño et al. [12] attribute the improvement to both the formation of additional calcium silicate hydrate (C–S–H) through pozzolanic reactions and the filler effect, in which fine ceramic particles occupy voids within the paste, resulting in pore refinement. As a result, mortars and concretes containing ceramic powders typically exhibit reduced total porosity and improved microstructural integrity, which in turn enhances their mechanical performance and durability.

While numerous studies have explored the rheological and mechanical behaviour of cements incorporating ceramic powders as SCMs, research on their durability performance remains limited. The available literature is sparse, offering only insights into a narrow range of durability aspects. In particular, most of the existing studies focus almost exclusively on permeability and resistance to chloride ingress, with virtually no attention given to other critical durability parameters.

Meena et al. [13], in their review, cite only a limited number of studies investigating the durability of cements containing ceramic powders as SCMs. Moreover, these few studies predominantly focus on permeability and resistance to chloride ingress, while other essential aspects of durability are largely neglected. In particular, no research has been reported on the performance of ceramic–SCM systems under extreme temperature conditions, such as high-temperature exposure or freezing. One of the few available studies addressing thermal durability is that of AlArab et al. [14], who performed freeze–thaw resistance tests on mortars incorporating porcelain powder. Their results indicated a reduction in frost resistance with increasing porcelain powder content, which they attributed to an increase in the overall porosity of the mortar matrix. Conversely, Kulovaná et al. [15] observed very good frost resistance in samples containing ceramic powder. These differences in results may stem from the types of ceramics used. The porcelain is fired at higher temperatures than the bricks and roof tiles studied by Kulovaná et al., which promotes the recrystallisation of metakaolin into mullite. This transformation reduces pozzolanic activity, resulting in a less dense matrix and lower freeze–thaw resistance.

All durability studies conducted to date have consistently shown that the incorporation of ceramic powders in cementitious materials leads to improved resistance to chemical aggression. El-Dieb et al. [11] found that the addition of 40% ceramic powder reduced chloride ion penetration at 28 days by 83% compared to ordinary concrete. Kannan et al. [5] obtained similar results, reporting an 89% reduction in chloride penetration at 28 days with a 40% replacement level. The results of Wang et al. [16] indicated that a 40% ceramic powder content reduces chloride penetration at 28 days by approximately 72%. Chen et al. [17], however, reported a 27.3% reduction in chloride penetration at a 10% substitution rate, with lower effectiveness observed at higher replacement levels. Tremiño et al. [12] reported a noticeable decrease in chloride diffusion after 4 years in mortar containing 20% brick powder compared to OPC mortar. Kulovaná et al. [15] reported excellent resistance of concrete containing 20% ceramic powder to magnesium and ammonium chloride solutions with concentrations of 17.76 and 2.97 g/L, respectively, as well as to a sodium sulphate solution (14.79 g/L) and a hydrochloric acid solution (10^−3^ mol/L). Zita et al. [18] demonstrated that concrete containing 24% ceramic powder exhibited a ≥48% reduction in the chloride diffusion coefficient compared to Portland cement concrete and showed good performance against sulphate attack in a 0.352 mol/L Na_2_SO_4_ solution. Mohammadhosseini et al. [19] investigated cement mortars incorporating 40% ceramic powder as a cement replacement and 100% recycled ceramic fine aggregate. After 18 months of curing in a 5% NaCl solution, the average penetration depth in the ceramic-containing mortar was 67% lower compared to that in OPC mortar. Exposure to a 5% Na_2_SO_4_ solution for up to 18 months resulted in compressive strength reductions of 41.1% and 16.8% for OPC and ceramic mortars, respectively.

According to the cited sources, this enhanced durability is primarily attributed to the improvement in the microstructure of the cement matrix, particularly the refinement of the pore structure. Additionally, the improved resistance to chemical attacks is also explained by the reduced presence of portlandite (Ca(OH)_2_) in the system due to its consumption during pozzolanic reactions. Ikotun et al. [20], in their review, highlight the limited research on the durability of cementitious materials incorporating ceramic SCMs. However, the available studies indicate that durability properties are improved due to matrix densification resulting from pozzolanic reactions.

Recently, considerable attention has been paid to ternary calcined clay–limestone–cement (LC3) binders, developed by the EPFL research team (Switzerland) and successfully tested in industrial trials in Cuba and India [21]. Mañosa et al. [22] conducted an extensive bibliographic analysis, highlighting the growing interest in LC3 research. This blended cement formulation, combining Portland cement with metakaolin and limestone filler, is attractive due to its excellent chemical resistance and potential for significant energy savings and reduced CO_2_ emissions as noted by Ijas et al. [23] and by Sharma et al. [24] in their extensive reviews.

While ceramic powders have been extensively studied in binary cement systems, their application in ternary LC3 systems remains largely unexplored. Binary systems (cement + ceramic and cement + limestone) typically show improvement in mechanical properties at relatively low cement replacement rate, whereas ternary LC3 systems (cement + ceramic + limestone) offer potential improvement at higher replacement through synergetic effect and calcium carboaluminates formation [25]. However, no studies have directly compared the durability performance of these two approaches.

De Matos et al. [26] investigated the rheological and mechanical properties of LC3, in which natural calcined clay was partially or completely replaced by ceramic waste powder. The results showed that the ceramic powder exhibited reactivity, albeit less than that of natural calcined clay. The formulations containing ceramic powder demonstrated slightly lower strength but improved flowability compared to those with calcined clay. Overall, both partial and complete replacement of natural calcined clay with ceramic waste powder proved to be an effective strategy for controlling the rheological properties of fresh LC3 and produced cements that met standard strength classes.

Mohit et al. [25] explored the mechanical strength and ASR of cement–ceramic–limestone mixtures. The optimal formulation of 65% cement, 20% ceramic powder, and 15% limestone filler showed compressive strengths within about 3% of the reference OPC mortar at all curing ages, while reducing ASR expansion by approximately 55%.

Marangu [27] examined the effects of 3% sulphuric acid attack on hydrated LC3 cement mortars containing 30% waste ceramic bricks as a substitute for calcined clay and 15% limestone filler. The study concluded that LC3 cement using waste ceramic bricks demonstrated improved mechanical properties and markedly higher resistance to sulphuric acid attack compared to OPC.

### 1.2. Scope, Novelty, and Relation to Previous Work

As shown in the literature review, existing studies on the durability of blended cements with ceramic SCMs have primarily focused on chemical resistance, especially chloride penetration, while the effects of extreme temperatures remain largely unexplored. A similar gap exists for LC3 systems, and no published studies appear to have examined the durability of ternary cement–ceramics–limestone filler systems.

The present study addresses this gap by evaluating the durability of mortars with reduced Portland cement clinker content through the incorporation of recycled ceramics used both as pozzolanic SCM in binary systems and as a substitute for calcined clay in LC3-type binders. The work focuses on durability criteria that are underrepresented in the literature for ceramic waste binders, including freeze–thaw resistance, thermal degradation, and sulphuric acid attack. It also provides a direct comparison between binary and LC3-type mortars, an approach rarely found in existing research. Furthermore, the study adapts the LC3 concept by substituting recycled ceramic demolition waste for industrial calcined clay, offering a more practical and energy-efficient alternative.

This work forms part of a larger project on the valorisation of demolition wastes as SCMs. It is related to an earlier study [28], which investigated pozzolanic activity, microstructure, rheology, and mechanical properties of mortars with waste terracotta and sanitary porcelain powders. In this study, only terracotta powder was considered because, unlike porcelain, it exhibited significant pozzolanic activity and enhanced mortar strength. Both particle size fractions, <125 μm and <63 μm, demonstrated good performance, with an optimal cement replacement level of 20%, which was selected for further investigation. The current paper provides original durability data, which are critical for assessing the feasibility of these binders in practice, given that low durability and incapacity to resist aggressive environments can lead to premature structural failure.

While some basic material property data from [28] are repeated for context in the Materials and Methods section, all durability test results given in the Results and Discussion section and related interpretations in this work are original. This separation was necessary to maintain focus and ensure that each paper met the length and scope expectations of a research article, while together they provide a comprehensive assessment of ceramic waste as a sustainable SCM.

This research demonstrates a circular economy approach to construction materials, where end-of-life ceramic products from demolition are transformed into value-added supplementary cementitious materials, simultaneously addressing waste management challenges and cement industry decarbonisation.

## 2. Materials and Methods

### 2.1. Materials

Ceramic roof tile waste was recovered from a landfill site located in Luxembourg. To simulate a process with minimal environmental impact and energy consumption, the waste was not pre-treated and was dried under natural conditions. The collected material was initially reduced to fragments of approximately 30 mm using a hammer and further processed with a jaw crusher to reduce particle size. Subsequently, the material was subjected to dry grinding in an impact mill fitted with a 2 mm mesh bottom screen. Two granulometric fractions were then prepared by sieving: particles passing through a 125 µm mesh were classified as fine ceramic powder (RT), while those passing through a 63 µm mesh were designated as ultrafine ceramic powder (RTU).

**Figure 1:**
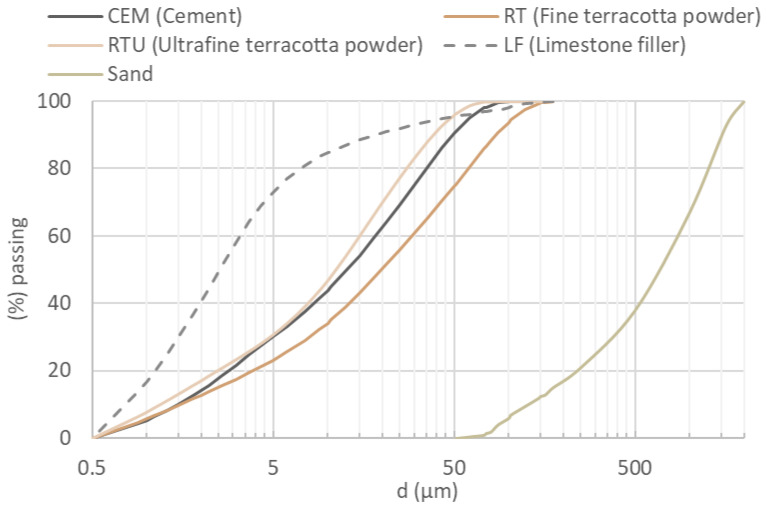
Particle size distribution of the used materials [28].

In addition to the processed ceramic waste, the materials employed in the mortar specimens design included Portland cement CEM I 52.5 R and limestone filler (LF), as well as CEN Standard Sand in accordance with EN 196-1 [29], and tap water. CEM I 52.5 R was selected because it contains no additives other than gypsum and provides rapid strength development, making it suitable for laboratory investigations. The physical properties of the powdered raw materials and the pozzolanic activity of ceramic powders measured by the Chapelle test are presented in Table 1. The granulometric compositions of the used materials are given in Figure 1. All these data were previously assessed in detail in the earlier study and are presented here for context, serving as a reference point for interpreting the observed durability performance of mortars containing terracotta powder.

The ceramic waste exhibited a combined content of silica, alumina, and iron oxides amounting in sum to 76.72%, exceeding the minimum threshold of 70% specified by the ASTM C618 [32] standard for pozzolanic materials. The complete chemical composition of the waste terracotta, cement, and limestone is provided in Table 2.

The main mineralogical phases of the waste ceramics were predominantly presented by quartz, feldspars, and an amorphous phase. The minor phases were presented by hematite and gypsum [28].

### 2.2. Packing Density

To assess the particle packing density of cement mortars, a modified Andersen and Andersen (A&A) method was employed [33]. This approach evaluates how closely the particle size distribution (PSD) of the studied mixture approximates an ideal theoretical distribution that provides maximum packing density.

First, the cumulative PSD of each mortar mixture was calculated from the weighted contributions of its individual components given in Figure 1 according to the following equation:

(1) $$P_{i}^{Mixture}=\frac{a\cdot P_{i}^{CEM}+b\cdot P_{i}^{RT}+c\cdot P_{i}^{RTU}+d\cdot P_{i}^{LF}+e\cdot P_{i}^{Sand}}{a+b+c+d+e},$$

where *P_i_* is the cumulative percentage of particles corresponding to point *i* (CEM is cement; RT is fine terracotta powder; RTU is ultrafine terracotta powder; LF is limestone filler); *a*, *b*, *c*, *d* are the mass fractions of the corresponding components in the binder (*a* + *b* + *c* + *d* = 1); *e* is the mass fraction of sand in the total mixture (*e *= 3).

The ideal PSD was then generated using the A&A model:

(2) $$P_{i}^{Ideal}=\frac{D_{i}^{q}-D_{min}^{q}}{D_{max}^{q}-D_{min}^{q}}\cdot 100,$$

where *D_i_* is the particle size corresponding to point *i*; *D_min_* and *D_max_* are the minimum and maximum particle sizes in the system; *q* is the distribution modulus. In this study, *q* = 0.37 was used, consistent with values reported in the literature for achieving optimal packing [34,35,36].

To evaluate the packing quality of the mortar mixtures, the degree of similarity *S* between the real PSD and the ideal PSD was calculated using the following equation:

(3) $$S=\frac{1}{n}\sum_{i=1}^{n}\left(1-\frac{\left|P_{i}^{Ideal}-P_{i}^{Mixture}\right|}{P_{i}^{Ideal}}\right),$$

where *n* is the number of particle size intervals considered.

A similarity score *S* closer to 1 indicates that the particle size distribution of the mixture closely matches the ideal distribution, suggesting higher packing density.

### 2.3. Preparation of Mortar Specimens

Experimental testing was carried out on 40 × 40 × 160 mm prismatic mortar specimens prepared in accordance with ISO 679:2009 [37]. All mixes were designed with constant water-to-binder and sand-to-binder ratios of 0.5 and 3.0, respectively. In the reference and ceramic powder-based mortars, 20% of the Portland cement was replaced by mass. For the LC3-type formulations, a 30% binder replacement was adopted, consisting of 20% terracotta powder combined with 10% limestone filler. Mortar constituents were mixed in a laboratory mixer following the sequence specified in ISO 679:2009. The fresh mortar was placed in oiled prismatic moulds in two layers, each compacted using a jolting table. After casting, the moulds were covered with a glass sheet for 24 h, after which the specimens were demoulded, covered with a plastic film to prevent moisture loss, and stored at 20 °C for 28 days before testing.

**Figure 2:**
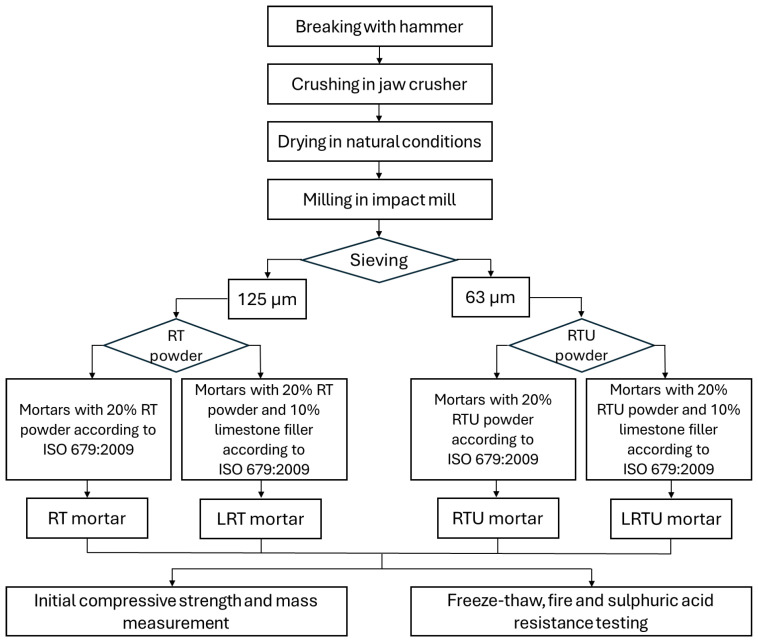
Specimen preparation flowchart.

The full mix proportions and mean compressive strength of mortars at 28 and 90 days of curing are presented in Table 3. The compressive strength of the mortars was previously assessed in detail in the earlier study and is presented here for context, serving as a reference point for evaluating strength losses after the durability tests. The specimen preparation flowchart is shown in Figure 2.

### 2.4. Capillary Absorption

**Figure 3:**
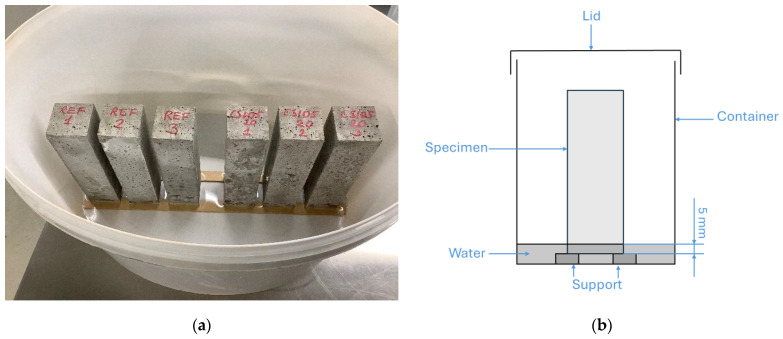
Experimental arrangement for the capillary water uptake test: (**a**) specimens setup during testing; (**b**) schematic representation of the arrangement.

European standard EN 480-5 [38] was used for the capillary absorption measurement. Three prismatic samples were prepared for each formulation and subjected to testing after a 28-day curing period. Prior to testing, all specimens were oven-dried at 50 °C until mass stabilisation was achieved, after which their dry masses were recorded. This drying temperature was chosen to prevent the decomposition of cement hydration products, which begins to occur at temperatures close to 100 °C. Dried specimens were positioned vertically with a 40 × 40 mm face in contact with water, to be plunged at 5 mm depth, as illustrated in Figure 3. The 5 mm water depth was marked directly on the specimens. During testing, the water level was visually aligned with these marks and maintained to ensure a consistent immersion depth. To minimise evaporation, the container was sealed during the test.

Mass gain due to water uptake was recorded after 0.25, 0.5, 1, 2, 4, 6, 24, 48, and 168 h of testing. Before each weighing, excess water was gently removed from the surface with absorbent material.

The capillary absorption coefficient (*C_a_*), expressed in g/mm^2^, was calculated using the following equation:

(4) $$C_{a}=\frac{M-M_{0}}{1600},$$

where *M*_0_ is the initial dry mass (g), *M* is the mass after water absorption (g), and 1600 mm^2^ is the surface area of the immersed face.

For data analysis and graphical presentation in Section 3.2. Capillary Absorption, mean values and standard deviations derived from three specimens per mixture are reported.

### 2.5. Drying Shrinkage

**Figure 4:**
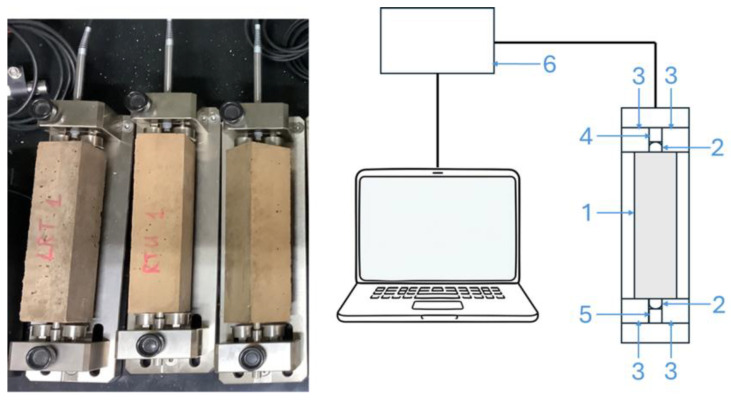
Schematic of the apparatus used for drying shrinkage measurements: (1) mortar specimen; (2) integrated attachment pins; (3) ball-bearing rollers; (4) digital displacement gauge; (5) stationary pin; (6) signal processor.

Drying shrinkage was evaluated in accordance with EN 12617-4 [39], with modifications introduced to the measurement schedule and overall test duration. Three prismatic specimens (40 × 40 × 160 mm) were prepared per mortar type, cast in moulds equipped with embedded metal pins at both ends to enable length change monitoring. After 24 h of curing, the samples were demoulded and transferred onto a Type C Shrinkage Measurement System (Testing Bluhm & Feuerherdt GmbH, Berlin, Germany), as depicted in Figure 4.

Length variations were recorded using a digital displacement sensor fitted with LVDT transducers (accuracy: 0.2 μm; resolution: 0.31 μm; range: 5 mm) and a conical contact tip. One end of the specimen remained horizontally fixed against a reference pin, while the displacement gauge contacted the opposite end to detect axial deformation over time. Shrinkage readings were taken at 2 h intervals over a 90-day period using a signal processing unit from Schleibinger Geräte.

Throughout the test, relative humidity and ambient temperature were recorded.

### 2.6. Freeze–Thaw Resistance

A non-standardized protocol [40] was employed to comparatively evaluate the durability of mortar specimens in freeze–thaw conditions. Prismatic samples (40 × 40 × 160 mm), previously cured for 28 days, were dried in a drying cabinet at 50 °C until mass stabilisation and weighed to determine dry mass before durability testing. Following this, specimens were submerged in water inside sealed containers and put in a programmable freezing unit.

The specimens were cooled to −20 °C, held at this temperature for 11 h 50 min, and then warmed back up to +20 °C. The cooling and heating rate was 4 °C per minute. The duration of one complete cycle was 24 h. In total, 28 cycles were applied, consistent with previous research on cement-based materials, to balance experimental feasibility and sensitivity to performance differences among mixtures.

Upon completion of the cycling sequence, the specimens were redried to a constant mass and reweighed. Compressive strength was then measured for each mortar type. Resistance to freeze–thaw action was quantified as the change in mass and strength before and after exposure, expressed as a percentage.

Average values from six specimens were reported along with corresponding standard deviations. Since there is no established method for calculating the standard deviation of a percentage value derived from two independent sets of measurements, a cross-combination approach was applied. Specifically, all possible pairwise combinations between the pre- and post-exposure specimens were used to compute 36 individual strength loss percentages (6 × 6 combinations). The standard deviation of these values was then calculated to represent the spread. While this approach inevitably leads to a higher standard deviation due to capturing all potential differences between independent specimens, it reflects the natural scatter in compressive strength results and allows for a more comprehensive representation of variability in the derived loss values.

### 2.7. Fire Resistance

Before testing, the initial mass of the samples was measured, then placed in a muffle furnace and heated to 200 °C, 300 °C, 500 °C, or 900 °C for 2 h. The temperature increase rate was 10 °C per minute, and the samples cooled down naturally in a closed furnace chamber. The weight of the samples was measured immediately after cooling to ambient temperature to assess the degree of degradation. Fire resistance was evaluated by calculating the reduction in mass and mechanical strength after exposure, expressed as a percentage.

For each thermal condition and mortar type, six replicate specimens were tested. The results are presented as average values with corresponding standard deviations, following the statistical procedure described previously for freeze–thaw resistance evaluation.

### 2.8. Sulphuric Acid Resistance

After 28 days of curing, cement mortar specimens measuring 40 × 40 × 160 mm were dried in a drying chamber at 50 °C until the stabilisation of mass, which was then measured before immersion of the specimens in a 1% sulphuric acid solution for 90 days. Every 30 days, the solution was renewed to maintain constant acidity throughout the test.

At the end of the exposure period, mortar bars were rinsed with tap water, dried in a drying chamber at 50 °C to remove moisture, weighed, and tested for compressive strength.

Resistance to sulfuric acid was expressed as percentage changes in weight and strength, calculated relative to initial values. Analysis was performed on six replicates per mortar formulation, with mean values and standard deviations reported following the approach described for freeze–thaw resistance tests.

### 2.9. Statistical Analysis

Multivariate analysis of variance (MANOVA) was employed in this study to evaluate the influence of multiple independent variables on a set of durability-related response variables simultaneously. This statistical technique is particularly appropriate when several dependent variables are interrelated, allowing for a more comprehensive understanding of their collective variation. In this case, compressive strength losses after freeze–thaw, high temperatures, and 1% sulphuric acid exposure are interrelated because they are all influenced by common material characteristics such as microstructure, porosity, and binder composition, which govern the material’s behaviour under various environmental stressors. For instance, higher porosity or altered binder composition can simultaneously affect frost, fire, and acid corrosion resistances. Thus, MANOVA provides a way to evaluate the combined effects on these durability indicators, offering a more holistic view than separate univariate tests.

The analysis was conducted in the R programming environment. The dependent variables included freeze–thaw resistance, fire resistance at four temperature levels, and sulphuric acid resistance. These variables were selected as key indicators of mortar durability under various aggressive environmental conditions.

The independent variables considered were the specific surface area of the raw material powders, the presence or absence of limestone filler, drying shrinkage, and capillary absorption. These factors were chosen based on their expected impact on the microstructure, porosity, and overall stability of the cementitious matrix.

Statistical significance was assessed using *p*-values derived from the multivariate test statistics. While a significance threshold of 0.05 is commonly used, the main focus here was on comparing the *p*-values of different factors to determine which independent variables had the most substantial effect on the combined durability parameters. Smaller *p*-values indicate stronger evidence against the null hypothesis, suggesting a significant multivariate effect of the factor on the durability outcomes. Factors with *p*-values greater than 0.05 indicate weaker effects or no significant impact. By comparing these *p*-values, it was possible to assess the relative influence of each independent variable on the overall durability, highlighting their distinct contributions.

## 3. Results and Discussion

### 3.1. Packing Density

**Figure 5:**
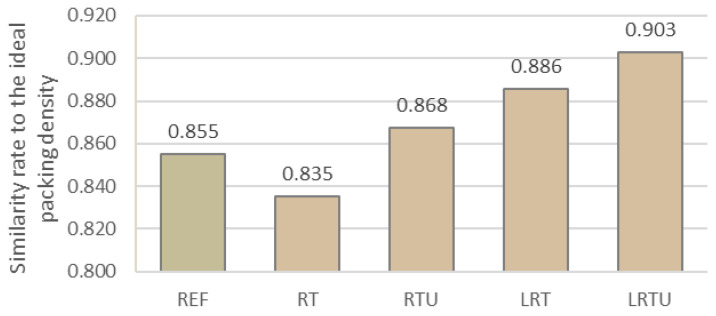
Packing densities of the mortar mixtures: REF—reference mortar; RT—mortar with fine terracotta powder; RTU—mortar with ultrafine terracotta powder; LRT—LC3 mortar with fine terracotta powder; LRTU—LC3 mortar with ultrafine terracotta powder.

The calculated packing densities for the different binder systems, determined using the modified Andersen and Andersen method and expressed in similarity score, are summarized in Figure 5.

The RT system, containing fine terracotta powder, displayed a slightly reduced packing density (0.835), suggesting that the particle size distribution of this ceramic powder does not completely complement that of cement. By contrast, the use of ultrafine terracotta powder led to a notable increase in packing density (0.868). This enhancement can be attributed to the ability of finer particles to effectively occupy voids between coarser grains, thus improving the overall packing structure.

A further increase was observed in systems incorporating limestone filler. The LRT binder contained limestone filler and fine terracotta powder, achieving a packing density of 0.886. The LRTU system, where the ultrafine ceramic powder was used, reached the highest packing density of all tested mixtures (0.903). These results clearly demonstrate that combining powders of different particle size ranges significantly enhances the packing efficiency. The synergy between the particle size distributions of sand, cement and ceramic, and limestone powders promotes the formation of a denser granular skeleton, reducing interparticle voids and enhancing material compactness, which is expected to improve durability.

### 3.2. Capillary Absorption

**Figure 6:**
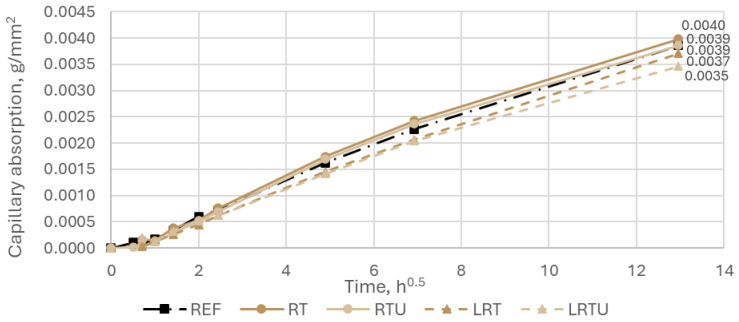
Capillary absorption of mortar specimens: REF—reference mortar; RT—mortar with fine terracotta powder; RTU—mortar with ultrafine terracotta powder; LRT—LC3 mortar with fine terracotta powder; LRTU—LC3 mortar with ultrafine terracotta powder.

**Figure 7:**
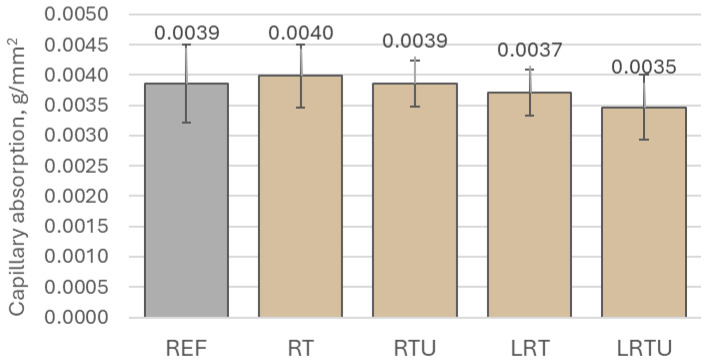
Capillary absorption of mortar specimens after 7 days of contact with water: REF—reference mortar; RT—mortar with fine terracotta powder; RTU—mortar with ultrafine terracotta powder; LRT—LC3 mortar with fine terracotta powder; LRTU—LC3 mortar with ultrafine terracotta powder.

The capillary absorption behaviour of the tested mortar mixtures is illustrated in Figure 6 and Figure 7. Among all specimens, the mortar incorporating fine terracotta powder RT exhibited the highest water uptake, reaching 0.0040 ± 0.00052 g/mm^2^ after 7 days, which may be explained by the lower specific surface area of the RT powder, resulting in a more porous microstructure. The RTU specimen showed a capillary absorption of 0.0039 ± 0.00038 g/mm^2^, identical to that of the reference mortar (0.0039 ± 0.00064 g/mm^2^). The addition of limestone filler led to a modest yet consistent decrease in water absorption in both corresponding mortars. Specifically, the LRT specimen showed an absorption of 0.0037 ± 0.00038 g/mm^2^, while the LRTU mortar exhibited the lowest value at 0.0035 ± 0.00053 g/mm^2^. This reduction is likely due to the improved packing density associated with the finest granulometry of the limestone powder, which enhanced matrix densification and led to a decrease in porosity. However, the differences in absorption values across all mixtures fall within the range of experimental variability, as indicated by the overlapping standard deviations. Therefore, despite a general trend of reduced absorption with denser particle packing, these differences may be considered statistically insignificant.

Although both the compressive strength (Table 3) and capillary water absorption results demonstrated improvement with the use of ceramic powders possessing a higher specific surface area, no clear correlation was observed between compressive strength and water absorption. Notably, the reference mortar, despite exhibiting the highest compressive strength, showed greater capillary absorption than the LRT and LRTU mortars, which exhibited lower strength values. This phenomenon can be attributed to the mechanically weaker binder matrix in the specimens containing ceramic waste powders, particularly in the LC3-based mortars, despite the improvement in particle packing density. In systems incorporating ceramic powders and limestone, the pozzolanic activity and the formation of carboaluminate phases are slower processes relative to the hydration of clinker phases [41]. Therefore, despite a denser structure, the percolation of strong chemical bonds within the matrix remains incomplete at 28 days, leading to comparatively lower mechanical strength but reduced capillary sorptivity. Other researchers have also reported results indicating no direct correlation between the compressive strength and water absorption of samples containing ceramic powders [42,43]. Pacheco-Torgal and Jalali [43] attribute this to enhanced hydration during water absorption testing, resulting from the additional water supply.

### 3.3. Drying Shrinkage

Drying shrinkage refers to the volume reduction of hardened cementitious materials as they lose moisture to the surrounding environment. This phenomenon primarily occurs due to water loss from the gel pores, which are extremely fine pores (typically <10 nm) within the C-S-H gel, which constitutes the primary strength-giving phase in hydrated cementitious systems. As water evaporates from these nanoscale pores, internal stresses develop within the matrix, leading to volumetric contraction and microcracks formation.

**Figure 8:**
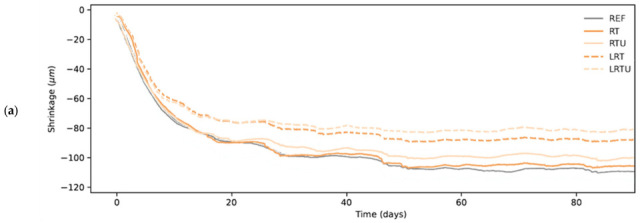

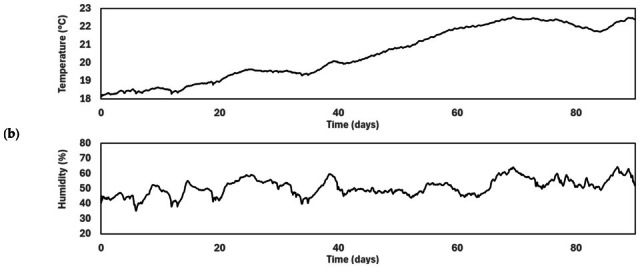
Shrinkage development in mortars during 90-day drying period: (**a**) drying shrinkage curves: REF—reference mortar; RT—mortar with fine terracotta powder; RTU—mortar with ultrafine terracotta powder; LRT—LC3 mortar with fine terracotta powder; LRTU—LC3 mortar with ultrafine terracotta powder; (**b**) temperature and relative humidity curves.

Figure 8 illustrates the development of drying shrinkage in the mortars throughout the 90-day test duration. Drying shrinkage increased rapidly up to about 10 days, slowed between 10 and 30 days, continued at a lower rate until approximately 50 days, and then stabilised. This trend can be explained by initial rapid moisture evaporation from the cement matrix and accelerated hydration, followed by gradual pore refinement due to hydration product formation, which reduced moisture loss. As available water became depleted, shrinkage development decelerated and eventually ceased, consistent with the mechanisms reported by Nasir et al. [44].

Throughout the testing duration, the LC3-type mortar specimens exhibited the lowest drying shrinkage. The RT curve closely matched the reference sample curve for approximately two months, after which it displayed slightly lower shrinkage, although remaining very close. The RTU curve followed the reference and RT curves up to around 20 days, subsequently exhibiting reduced shrinkage until the end of the experiment. Similar trends have been noted by other authors, who documented decreased shrinkage in mixtures with ceramic components [9,11].

After 90 days of drying, the shrinkage values recorded for the REF, RT, RTU, LRT, and LRTU specimens were 112 μm/m, 105 μm/m, 100 μm/m, 88 μm/m, and 80 μm/m, respectively. The observed shrinkage decrease, particularly in RTU- and limestone-containing mixtures, is likely linked to enhanced particle packing due to the finer granulometry of the ultrafine ceramic powder and limestone filler.

Fine limestone particles act as fillers, occupying spaces between cement grains. By optimising the particle size distribution and enhancing packing density, limestone fillers reduce matrix porosity. Moreover, because limestone partially replaces clinker without significantly contributing to additional C-S-H formation, it does not substantially increase the fine gel pore volume.

Interestingly, despite exhibiting lower capillary absorption, the REF specimens showed slightly higher drying shrinkage than the RT specimens. This can be explained by the fact that drying shrinkage is mainly influenced by the gel pore network [45], while the mechanism of capillary absorption in cementitious materials is very complex and depends not only on pore size, but also on pore connectivity and swelling due to the interaction of the C–S–H gel with water [46,47]. Therefore, a material can exhibit low absorption but still experience significant shrinkage if the gel pore volume remains relatively high.

### 3.4. Freeze–Thaw Resistance

**Figure 9:**
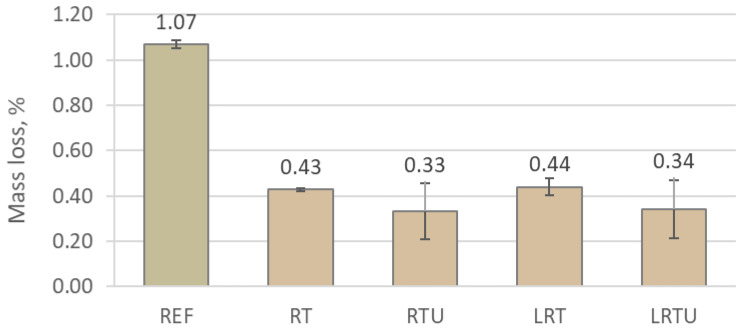
Mass loss of mortars after 28 freeze–thaw cycles: REF—reference mortar; RT—mortar with fine terracotta powder; RTU—mortar with ultrafine terracotta powder; LRT—LC3 mortar with fine terracotta powder; LRTU—LC3 mortar with ultrafine terracotta powder.

**Figure 10:**
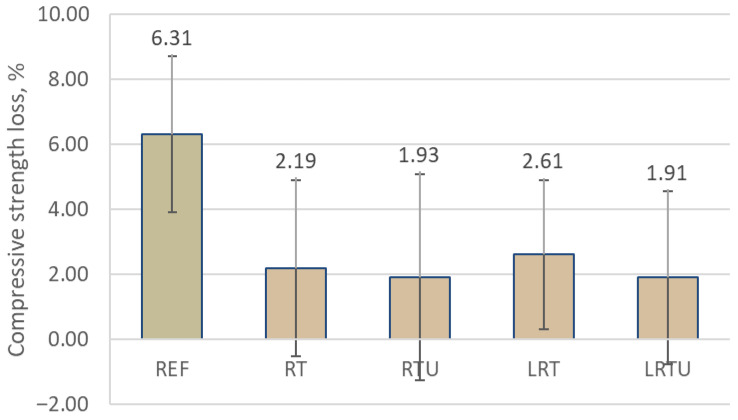
Compressive strength loss of mortars after 28 freeze–thaw cycles: REF—reference mortar; RT—mortar with fine terracotta powder; RTU—mortar with ultrafine terracotta powder; LRT—LC3 mortar with fine terracotta powder; LRTU—LC3 mortar with ultrafine terracotta powder.

The freeze–thaw resistance results, expressed as mass loss and compressive strength loss after 28 cycles, are presented in Figure 9 and Figure 10. The results demonstrated a clear improvement in the mortars incorporating ceramic waste powders compared to the reference mortar. The REF specimen exhibited a mass loss of 1.07% and a strength loss of 6.31%, whereas the modified mortars (RT, RTU, LRT, and LRTU) showed significantly lower values. The best performance was observed for RTU and LRTU specimens, which exhibited mass losses of 0.33% and 0.34%, and strength losses of 1.91% and 1.93%, respectively.

It seems that increasing the fineness of the ceramic powder positively affects the resistance of the mortars to freeze–thaw cycles. However, considering the relatively high measurement uncertainties (standard deviations), the absence of a definitive effect of powder fineness cannot be entirely excluded. The addition of limestone filler did not appear to exert a substantial influence on freeze–thaw resistance under the testing conditions. A correlation was observed between the compressive strength (Table 3) and the subsequent freeze–thaw performance of the modified mortars. Specimens that achieved higher early age compressive strengths generally exhibited smaller decreases in mass and mechanical strength after freeze–thaw test. This observation suggests that a stronger matrix better resists the internal stresses generated by the expansion of freezing water within the pore structure.

Nevertheless, the REF specimen, despite having the highest initial strength, showed significantly greater mass and strength losses than the modified specimens. Given that the REF mortar also exhibited the highest drying shrinkage, it may be inferred that shrinkage-induced microcracking contributed to its reduced durability. Therefore, drying shrinkage emerges as an important factor influencing freeze–thaw resistance, alongside compressive strength and capillary adsorption. Its role has been specifically highlighted and investigated by Miao et al. [48].

Moreover, the continuous immersion of specimens in water during the freeze–thaw cycles likely provided a favourable environment for pozzolanic reactions to occur in the mortars containing ceramic powders. Pozzolanic reactions between the reactive aluminosilicate phases in the ceramic waste and the calcium hydroxide released during cement hydration require the presence of water to progress. The prolonged availability of moisture during the durability testing could have facilitated further formation of additional calcium silicate hydrate and other secondary binding phases. This late-stage reaction would contribute to further densification of the matrix, refinement of the pore structure, and a consequent increase in durability. Additionally, the formation of new hydration products may have partially sealed existing microcracks and pores, promoting self-healing phenomena that further improved the resistance of the mortars to freeze–thaw damage.

Thus, freeze–thaw resistance can be regarded as a multifactorial property, influenced by water permeability, initial matrix strength, shrinkage-induced microcracks, and the extent of pozzolanic reactions. Additional factors such as the ionic composition of the pore solution, the differential thermal expansion coefficients of mortar constituents, and the overall composition of the cementitious matrix also affect freeze–thaw behaviour [49].

### 3.5. Fire Resistance

**Figure 11:**
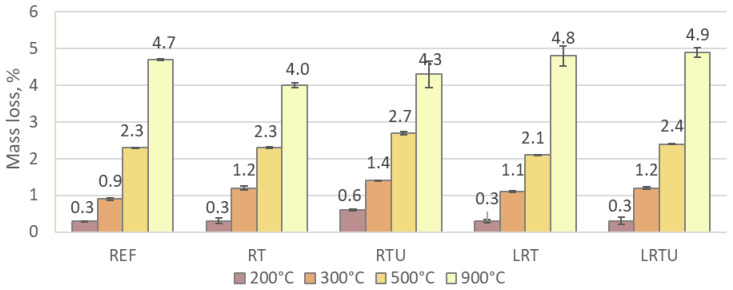
Mass loss of mortars after exposure to elevated temperatures: REF—reference mortar; RT—mortar with fine terracotta powder; RTU—mortar with ultrafine terracotta powder; LRT—LC3 mortar with fine terracotta powder; LRTU—LC3 mortar with ultrafine terracotta powder.

Mass changes of mortars following heating at 200 °C, 300 °C, 500 °C, and 900 °C are presented in Figure 11. Mass loss showed no notable variation at moderate temperatures (up to 500 °C) among all specimens, except for RTU. At 200 °C, the mass loss of the REF, RT, LRT, and LRTU specimens was approximately 0.3%, primarily associated with the dehydration of gypsum and ettringite, as well as the initial degradation of calcium silicate hydrates. At 300 °C, mass loss increased to between 0.9% and 1.2%, attributed to the decomposition of the calcium silicate hydrates. At 500 °C, mass loss reached values between 2.1% and 2.4% for the REF, RT, LRT, and LRTU specimens, corresponding to the onset of portlandite dissociation and further degradation of the C-S-H. The RTU mortar consistently exhibited slightly greater mass loss at each stage: 0.6% at 200 °C, 1.4% at 300 °C, and 2.7% at 500 °C.

Heating at 900 °C is associated with the full decomposition of C–S–H, portlandite, and calcite with consequent CO2 release. The corresponding mass losses were 4.7%, 4.0%, 4.3%, 4.8%, and 4.9% for REF, RT, RTU, LRT, and LRTU, respectively.

**Figure 12:**
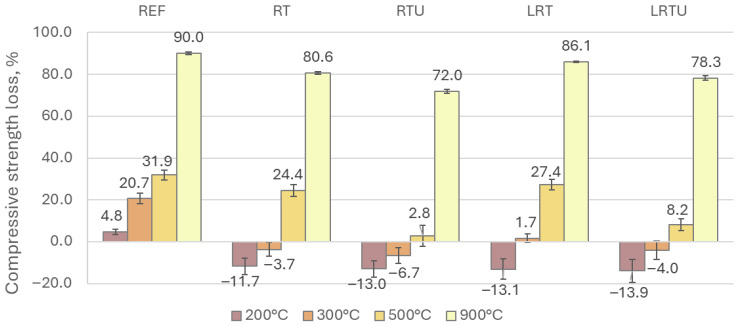
Compressive strength changes of mortars after exposure to elevated temperatures: REF—reference mortar; RT—mortar with fine terracotta powder; RTU—mortar with ultrafine terracotta powder; LRT—LC3 mortar with fine terracotta powder; LRTU—LC3 mortar with ultrafine terracotta powder.

The residual compressive strength values after thermal treatment are presented in Figure 12. The influence of ceramic SCMs was particularly evident at temperatures up to 300 °C. Upon heating to 200 °C and 300 °C, the reference specimen exhibited strength losses of 4.8% and 20.7%, respectively. In contrast, the RT, RTU, LRT, and LRTU specimens demonstrated strength gains of 11.7%, 13.0%, 13.1%, and 13.9% at 200 °C, respectively. The results at this temperature show a clear correlation with the drying shrinkage measurements, which is expected, as both phenomena are influenced by microstructural changes induced by water evaporation. At 300 °C, the RT, RTU, and LRTU specimens continued to exhibit strength gains of 3.7%, 6.7%, and 4.0%, respectively, while the LRT specimen showed only a minor strength loss of 1.7%. The observed strength enhancement at moderate temperatures is attributed to the acceleration of pozzolanic reactions, which are thermally activated. A similar phenomenon has been reported in [50,51,52] and in the review by Chong et al. [53], where it is explained by the increase in self-autoclaving, which in turn accelerates the rate of hydration of cement phases and pozzolanic reactions. These reactions promote the formation of additional cementitious phases and contribute to matrix densification, thereby improving mechanical performance.

**Figure 13:**
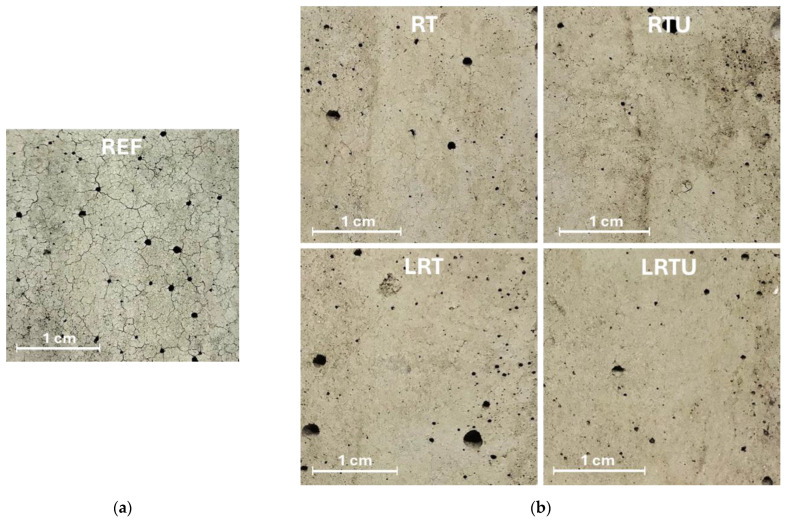
Surface texture of the mortar specimens after exposure at 900 °C: (**a**) reference mortar; (**b**) Mortars with ceramic powders: RT—mortar with fine terracotta powder; RTU—mortar with ultrafine terracotta powder; LRT—LC3 mortar with fine terracotta powder; LRTU—LC3 mortar with ultrafine terracotta powder.

At 500 °C, all specimens exhibited strength losses, 31.9%, 24.4%, 2.8%, 27.4%, and 8.2% for REF, RT, RTU, LRT, and LRTU, respectively, indicating superior performance for specimens incorporating the ultrafine ceramic powder (RTU). After exposure to 900 °C, substantial strength losses were observed across all mixes: 90.0%, 80.6%, 72.0%, 86.1%, and 78.3%, respectively. The best fire resistance was again recorded in the mortars containing RTU. The severe strength losses at 900 °C result from the water-releasing decomposition of calcium hydroxide and calcium silicate hydrates, CO_2_-releasing calcite decomposition, recrystallisation of calcium silicates into wollastonite, and the resulting cracking [54]. Ceramic-modified mortars retain more strength because the fired ceramic particles remain stable due to their quartz and feldspar content, whereas LC3 mixes are partly penalized by limestone decomposition, as also confirmed by Lin et al. [54]. Nevertheless, the LC3-type mortars still outperformed the reference specimen in terms of fire resistance. Visual observations further supported these findings: as shown in Figure 13, specimens containing ceramic powders exhibited fewer and finer surface cracks after exposure to 900 °C, whereas the OPC-based reference specimen displayed numerous wide cracks.

It is important to note that while thermal decomposition, such as dehydration and decarbonation, largely governs mass loss, changes in mechanical strength are influenced mostly by crack development due to phase transformations and microstructural alterations. Comparable mass loss was observed across all mortars, regardless of notable differences in residual strength. This suggests that internal characteristics such as mineralogical composition and pozzolanic reactivity, leading to the formation of additional cementitious phases, play a critical role in high-temperature mechanical stability.

Moreover, the same pattern was observed in fire resistance (Figure 12) and freeze–thaw resistance (Figure 10), indicating that both durability aspects may be influenced by shared properties, including porosity [55,56] and differences in thermal expansion coefficients between constituents [57,58].

### 3.6. Sulphuric Acid Resistance

**Figure 14:**
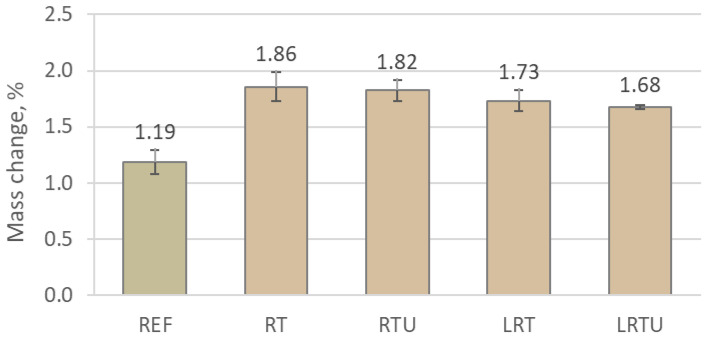
Mass change of mortar specimens after exposure to sulphuric acid solution: REF—reference mortar; RT—mortar with fine terracotta powder; RTU—mortar with ultrafine terracotta powder; LRT—LC3 mortar with fine terracotta powder; LRTU—LC3 mortar with ultrafine terracotta powder.

**Figure 15:**
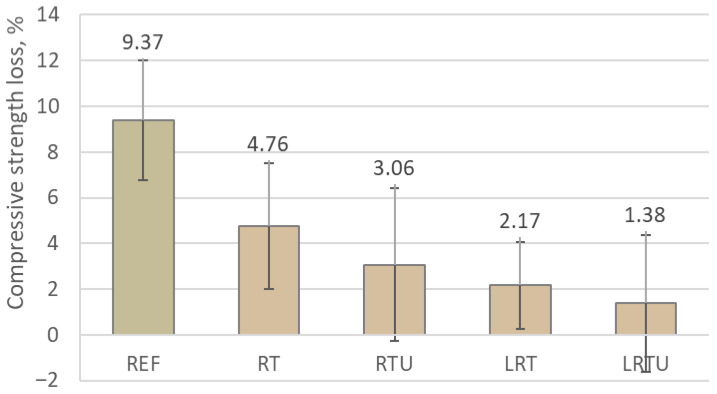
Loss in compressive strength of mortar specimens after immersion in sulphuric acid solution: REF—reference mortar; RT—mortar with fine terracotta powder; RTU—mortar with ultrafine terracotta powder; LRT—LC3 mortar with fine terracotta powder; LRTU—LC3 mortar with ultrafine terracotta powder.

The behaviour of the 28-day mortars under sulphuric acid exposure was investigated through mass variation and compressive strength retention after 90 days of immersion in a 1% H_2_SO_4_ solution, as shown in Figure 14 and Figure 15. Contrary to typical degradation trends, all formulations exhibited a net increase in mass. This phenomenon can be attributed to the ingress and reaction of sulphate ions with calcium-rich hydration products, causing the crystallisation of expansive secondary phases such as gypsum and ettringite. These compounds tend to precipitate within the outer pore network, resulting in a measurable gain in sample weight.

The specimens containing ceramic waste powders demonstrate a greater mass gain compared to the OPC mortar, showing mass gain of 1.19%, with RT, RTU, LRT, and LRTU achieving values of 1.86%, 1.82%, 1.73% and 1.68%, respectively. This greater mass gain may be attributed to the improved retention of the solid structure in the modified mortars after acid exposure. In OPC mortar, the higher content of portlandite, which dissolves readily under acidic conditions, promotes surface erosion and material loss, thereby reducing the net mass gain.

Changes in specimen mass are not always indicative of long-term performance in aggressive environments. The concurrent processes of ion exchange, including the incorporation of hydrogen and sulphate ions and the leaching of aluminium and calcium ions from the matrix, complicate interpretation [59,60]. The balance between these opposing mechanisms is governed by the exposure conditions, particularly acid concentration and duration [61,62,63].

In contrast to mass gain, the residual compressive strength provides a more reliable indicator of chemical durability. Notably, mortars containing ceramic additions exhibited superior strength retention compared to the control mix. While the reference suffered a 9.37% reduction in compressive strength, values for the alternative binders ranged from 4.76% (RT) to as low as 1.38% (LRTU). Similar results were reported by other researchers [4,64], who explained this improvement by pore refinement. It may also be attributed to the consumption of portlandite during pozzolanic reactions, which reduces the amount of acid-sensitive phases within the mortar, as observed in the case of sulphate attack [18].

Notably, specimens containing limestone filler exhibited the lowest strength losses. Additionally, an increase in the fineness of the ceramic powder further enhanced resistance to sulphuric acid attack. This can be explained by the improved pozzolanic reactivity of ultrafine powders, the greater portlandite-forming components dilution by higher clinker substitution in LC3-type mortars, and the reduction in permeability due to improved packing density.

### 3.7. Statistical Analysis

The multivariate analysis of variance MANOVA approach enabled the assessment of the extent to which each independent variable influences the durability outcomes, providing a clearer understanding of their relative contributions. While graphical comparisons indicated that all independent variables had an effect on the durability parameters, MANOVA allowed for a more precise quantification of the magnitude and statistical significance of each factor’s influence.

The results of the multivariate analysis of variance are summarised in Table 4. The Pillai’s trace values and associated F-statistics demonstrate notable differences in the strength of the effects exerted by each independent factor on the set of dependent durability variables.

In MANOVA, Pillai’s trace is a test statistic used to evaluate how much variation in the combined dependent variables can be attributed to each independent factor. The closer the value is to 1 (or higher, in the case of multiple levels), the stronger the effect. The approximate F-value measures the strength of the relationship and is used to test the statistical significance of this effect. A higher F-value generally means a more pronounced impact of the factor. The *p*-value indicates whether this effect is likely to be genuine or due to random chance; values below 0.05 are typically considered statistically significant. Together, these statistics help determine not only whether a factor has an effect, but how strong and reliable that effect is.

Below is an interpretation of the results showing the influence of each parameter ranked in order of decreasing effect size.

Among the analysed factors, ceramic powder fineness exerted the strongest multivariate effect (Pillai = 0.994, Approx. F = 385.80, *p* = 3.91 × 10^−8^), indicating an overwhelmingly dominant influence. This result underscores the critical role of particle size and surface area of pozzolan in affecting the durability characteristics of cementitious composites.

Limestone filler also exhibited a very strong effect (Pillai = 0.9739, Approx. F = 86.90, *p* = 6.66 × 10^−6^), confirming its significant multivariate contribution. Although slightly lower than the influence of fineness, the effect of limestone filler was still pronounced, highlighting its relevance in modifying the hydration process and microstructural development.

Capillary adsorption demonstrated a similarly strong and statistically significant multivariate influence (Pillai = 0.9730, Approx. F = 83.95, *p* = 7.49 × 10^−6^), which suggests that this parameter has a considerable impact on overall durability performance, likely due to its role in both chemical degradation and freeze–thaw damage mechanisms.

Shrinkage also displayed a strong and statistically significant multivariate effect (Pillai = 0.9663, Approx. F = 66.94, *p* = 1.61 × 10^−5^), supporting the notion that volumetric instability introduces internal microcracking, which can compromise durability over time.

Finally, packing density showed a notable effect as well (Pillai = 0.9297, Approx. F = 30.84, *p* = 2.09 × 10^−4^), indicating that more efficiently packed granular structures contribute meaningfully to the durability of the composites. This influence is attributed to improved matrix densification and reduced porosity.

A multivariate analysis of variance was conducted as a complementary tool to assess the combined influence of several mixture parameters on the durability-related properties of mortars. Given the relatively small dataset (*n* = 15), the statistical analysis remains limited in terms of formal inference. These findings should be considered preliminary and warrant validation with larger experimental designs.

While the MANOVA analysis provides valuable insights into factor interactions, the relatively small dataset (*n* = 15) limits the statistical power for detecting subtle effects. The significant factors identified (*p* < 0.05) represent robust effects that emerge despite the sample size constraints. These findings should be considered preliminary and warrant validation with larger experimental designs.

## 4. Conclusions

The results of this study provide direct evidence of the practical durability of mortars incorporating ceramic demolition waste, supporting its adoption in construction practice. The following conclusions were drawn:

1. Capillary absorption and drying shrinkage were reduced with increased ceramic powder fineness and limestone filler incorporation, due to enhanced matrix compaction. After 7 days, the lowest water uptake (0.0035 g/mm^2^) was observed for LRTU, compared to 0.0039 g/mm^2^ for the reference. Shrinkage after 90 days decreased from 112 μm/m (REF) to 80 μm/m (LRTU).
2. Freeze–thaw resistance improved in all modified mortars, with compressive strength losses reduced from 6.31% (REF) to between 1.91% and 2.61% in the alternative mixes. Increased ceramic powder fineness positively affected freeze–thaw resistance, while limestone filler had minimal impact under the tested conditions.
3. Mortars with ceramic powders demonstrated superior fire resistance, with strength gains at 200 °C and 300 °C attributed to thermally accelerated pozzolanic reactions. At 300 °C, the reference mortar lost 20.7% strength, while the mortar with ultrafine ceramic powder gained 6.7%. The addition of limestone filler slightly reduced fire resistance due to carbonate decomposition, but LC3-type mortars still outperformed the reference at temperatures up to 900 °C, where the reference lost 90% of strength compared to 72.0–86.1% for modified mortars.
4. Resistance to sulphuric acid improved with ceramic powder incorporation, further enhanced by limestone filler. Strength losses decreased from 9.37% in REF to 1.38% in LRTU, attributed to reduced permeability and portlandite dilution.
5. Statistical analysis (MANOVA) confirmed ceramic powder fineness as the most significant factor influencing durability (Pillai’s trace = 0.994, *p* = 3.91 × 10^−8^), followed by limestone filler (0.9739, *p* = 6.66 × 10^−6^) and packing density (0.9297, *p* = 2.09 × 10^−4^). Capillary absorption and shrinkage had significant but weaker effects.

In general, ceramic powders enhance mortar durability, with fineness being a key parameter. These findings have important practical implications:

- For development of standards: The superior durability of ceramic-modified mortars supports the inclusion of ceramic waste in cement standards, with recommended replacement levels of 20% for binary systems and 30% (comprising 20% ceramic and 10% limestone) for LC3 formulations.
- For reducing the cement industry’s carbon footprint: Since ceramic waste and limestone filler do not require thermal processing, replacing 20–30% of cement clinker may lead to a comparable reduction (20–30%) in CO_2_ emissions. However, an accurate assessment requires a life cycle analysis that accounts for the energy consumption associated with waste separation, transportation, and grinding.
- For waste management: Each tonne of cement produced could incorporate 200 kg of ceramic waste, diverting it from landfills and contributing to more sustainable waste utilisation.
- For practical applications: Given their reduced shrinkage and permeability, improved freeze–thaw resistance, superior fire resistance, and better chemical durability, the tested blended cements can be particularly suited for use in structural and non-structural masonry units, renders, repair mortars, and concretes exposed to aggressive environments, especially where both thermal stability and durability against moisture and chemical attack are required. However, further, more detailed and industrial-scale investigations are crucial before large-scale implementation.

## 5. Limitations and Outlook

This study provides an overall assessment of the durability performance of cements with ceramic SCMs. However, to achieve a more comprehensive understanding of the underlying degradation mechanisms, further investigations focusing on micro-structural and compositional changes during exposure to aggressive environments are necessary. Such analyses, including techniques like SEM and XRD, would elucidate the evolution of hydration products, phase transformations, and crack development, thereby strengthening the interpretation of durability outcomes.

The strength improvement observed in mortars with ceramic powder up to 300 °C, together with their reduced cracking at higher temperatures compared to the reference mortar, highlights the need for XRD analysis to identify the formation and decomposition of phases, and SEM/EDS to monitor crack development and locate the most vulnerable phases. In addition, XRD and SEM/EDS analyses of the outer layers of specimens exposed to sulphuric acid, conducted at specific intervals during immersion, would allow tracking of ettringite and gypsum formation and the associated stress development.

Future research integrating these microstructural insights with performance testing will contribute to optimising ceramic-based supplementary cementitious materials for sustainable cement applications.

## Figures and Tables

**Table 1:** Physical properties and pozzolanic activity of the powdered materials [28].

Property	CEM I	RT	RTU	LF
Specific gravity (g/cm^3^)	3.05	2.42	2.42	2.70
Blaine SSA (cm^2^/g)	5418	5239	6249	10,465
Ca(OH)_2_ fixed (mg/g) *	-	505	618	-

CEM I—cement; RT—fine terracotta powder; RTU—ultrafine terracotta powder; LF—limestone filler; SSA—specific surface area. * The minimum fixed Ca(OH)_2_ value indicating pozzolanic properties is 436 mg/g of material [30]; for metakaolin, the minimum value is 700 mg/g [31].

**Table 2:** Chemical composition in mass-% of the powdered materials [28].

Oxide	CEM I	Terracotta	Limestone
SiO_2_	16.07	50.64	8.54
Al_2_O_3_	3.91	17.66	1.96
Fe_2_O_3_	3.58	8.42	1.06
CaO	66.72	10.32	47.68
MgO	1.45	1.51	1.81
TiO_2_	0.37	1.08	0
MnO	0.08	0.19	0
Na_2_O	0.26	0.39	0.12
K_2_O	1.18	3.37	0.37
P_2_O_5_	0.39	0.23	0
SO_3_	3.88	3.28	0.1
LOI *	2.10	2.90	38.36

* Loss on ignition at 950 °C.

**Table 3:** Mix designs, fresh densities, and compressive strength of mortars [28].

**Mix**	**CEM I (kg/m^3^)**	**RT (kg/m^3^)**	**RTU (kg/m^3^)**	**LF (kg/m^3^)**	**Sand (kg/m^3^)**	**Water (kg/m^3^)**	**ρ_fresh_** **(kg/m^3^)**	**f_cm_,28d** **(MPa)**	**f_cm_,90d (MPa)**
REF	475	0	0	0	1426	238	2139	60.2	65.9
RT	371	93	0	0	1390	232	2084	51.8	64.2
RTU	378	0	94	0	1417	236	2125	54.5	68.2
LRT	323	92	0	46	1384	231	2075	44.0	53.7
LRTU	322	0	92	46	1380	230	2070	47.7	63.9

Columns: CEM I—cement; RT—fine terracotta powder; RTU—ultrafine terracotta powder; LF—limestone filler; ρ_fresh_—density of the fresh mortar; f_cm_,28d—mean compressive strength at 28 days of curing; f_cm_,90d—mean compressive strength at 90 days of curing. Rows: REF—reference mortar; RT—mortar with fine terracotta powder; RTU—mortar with ultrafine terracotta powder; LRT—LC3 mortar with fine terracotta powder; LRTU—LC3 mortar with ultrafine terracotta powder.

**Table 4:** MANOVA results.

Independent Factor	Df ^1^	Pillai ^2^	Approx F ^3^	Num Df ^4^	Den Df ^5^	Pr(>F) ^6^
Finess	1	0.994	385.80	3	7	3.91 × 10^−8^
Limestone filler	1	0.9739	86.90	3	7	6.66 × 10^−6^
Packing density	1	0.9297	30.84	3	7	2.09 × 10^−4^
Capillary adsorption	1	0.9730	83.95	3	7	7.49 × 10^−6^
Shrinkage	1	0.9663	66.94	3	7	1.61 × 10^−5^

^1^ *Df*—degrees of freedom associated with the independent factor. ^2^ *Pillai*—Pillai’s trace statistic, a multivariate test statistic indicating the proportion of explained variance; higher values suggest a stronger multivariate effect. ^3^ *Approx F*—approximate F-value, used to test the statistical significance of the multivariate effect; calculated based on the Pillai trace. ^4^ *Num Df*—numerator degrees of freedom for the F-distribution, related to the number of dependent variables. ^5^ *Den Df*—denominator degrees of freedom for the F-distribution, related to the residuals in the multivariate model. ^6^ *Pr(>F)*—*p*-value reflects the chance of the observed F-statistic under the null hypothesis; values below 0.05 suggest statistical significance.

## Data Availability

The original contributions presented in this study are included in the article. Further inquiries can be directed to the authors.

## Abbreviations

The following abbreviations are used in this manuscript:

SCMs	Supplementary cementitious materials
LC3	Limestone calcined clay cement
OPC	Ordinary Portland cement
C-S-H	Calcium silicate hydrate
ASR	Alkali–silica reaction
CO_2_	Carbon dioxide
CDW	Construction and demolition waste
RT	Fine terracotta powder/mortar with fine terracotta powder
RTU	Ultrafine terracotta powder/mortar with ultrafine terracotta powder
CEM I	Portland cement type I
LF	Limestone filler
PSD	Particle size distribution
A&A	Andreasen and Andersen (particle packing model)
SSA	Specific surface area
REF	Reference mortar
LRT	LC3 mortar with fine terracotta powder
LRTU	LC3 mortar with ultrafine terracotta powder
LVDT	Linear variable differential transformer
H_2_SO_4_	Sulphuric acid
MANOVA	Multivariate analysis of variance
Pillai	Pillai’s trace
Df	Degrees of freedom
Approx F	Approximate F-value
Num Df	Numerator degrees of freedom
Den Df	Denominator degrees of freedom
Pr(>F)	*p*-value associated with the F-statistic

## References

[1] Rispoli C., De Bonis A., Guarino V., Graziano S.F., Di Benedetto C., Esposito R., Morra V., Cappelletti P. (2019). The Ancient Pozzolanic Mortars of the Thermal Complex of Baia (Campi Flegrei, Italy). Constr. Build. Mater..

[2] Lavat A.E., Trezza M.A., Poggi M. (2009). Characterization of Ceramic Roof Tile Wastes as Pozzolanic Admixture. Waste Manag..

[3] Pitarch A.M., Reig L., Tomás A.E., Forcada G., Soriano L., Borrachero M.V., Payá J., Monzó J.M. (2021). Pozzolanic Activity of Tiles, Bricks and Ceramic Sanitary-Ware in Eco-Friendly Portland Blended Cements. J. Clean. Prod..

[4] Silveira V.A.L., de Resende D.S., Bezerra A.C.S. (2025). Sanitary Ware Waste in Eco-Friendly Portland Blended Cement: Potential Use as Supplementary Cementitious Material. CEMENT.

[5] Kannan D.M., Aboubakr S.H., EL-Dieb A.S., Reda Taha M.M. (2017). High Performance Concrete Incorporating Ceramic Waste Powder as Large Partial Replacement of Portland Cement. Constr. Build. Mater..

[6] Gonçalves J.P., Tavares L.M., Toledo Filho R.D., Fairbairn E.M.R. (2009). Performance Evaluation of Cement Mortars Modified with Metakaolin or Ground Brick. Constr. Build. Mater..

[7] Pang L., Liang H., Zhang D., Fang K. (2024). Combining Thermodynamic Modeling and Experiments to Characterize the Effect of Ceramic Polishing Powder in Cement-Based Materials. Constr. Build. Mater..

[8] (2020). Standard Test Methods for Measuring the Reactivity of Supplementary Cementitious Materials by Isothermal Calorimetry and Bound Water Measurements.

[9] Ebrahimi M., Eslami A., Hajirasouliha I., Ramezanpour M., Pilakoutas K. (2023). Effect of Ceramic Waste Powder as a Binder Replacement on the Properties of Cement- and Lime-Based Mortars. Constr. Build. Mater..

[10] Faldessai K., Lawande S., Kelekar A., Gurav R., Kakodkar S. (2023). Utilization of Ceramic Waste as a Partial Replacement for Cement in Concrete Manufacturing. Mater. Today Proc..

[11] El-Dieb A.S., Kanaan D.M. (2018). Ceramic Waste Powder: An Alternative Cement Replacement—Characterization and Evaluation. Sustain. Mater. Technol..

[12] Tremiño R.M., Real-Herraiz T., Letelier V., Ortega J.M. (2021). Four-Years Influence of Waste Brick Powder Addition in the Pore Structure and Several Durability-Related Parameters of Cement-Based Mortars. Constr. Build. Mater..

[13] Meena R.V., Jain J.K., Chouhan H.S., Beniwal A.S. (2022). Use of Waste Ceramics to Produce Sustainable Concrete: A Review. Clean. Mater..

[14] AlArab A., Hamad B., Assaad J.J. (2022). Strength and Durability of Concrete Containing Ceramic Waste Powder and Blast Furnace Slag. J. Mater. Civ. Eng..

[15] Kulovaná T., Vejmelková E., Keppert M., Rovnaníková P., Keršner Z., Černý R. (2016). Mechanical, Durability and Hygrothermal Properties of Concrete Produced Using Portland Cement-Ceramic Powder Blends. Struct. Concr..

[16] Wang S., Nguyen V.T., Xiu Z., Han W. (2023). Test Research on the Effect of Waste Ceramic Polishing Powder on the Compressive Strength and Chloride Penetration Resistance of Seawater Concrete. Constr. Build. Mater..

[17] Chen X., Zhang D., Cheng S., Xu X., Zhao C., Wang X., Wu Q., Bai X. (2022). Sustainable Reuse of Ceramic Waste Powder as a Supplementary Cementitious Material in Recycled Aggregate Concrete: Mechanical Properties, Durability and Microstructure Assessment. J. Build. Eng..

[18] Zito S.V., Cordoba G.P., Irassar E.F., Rahhal V.F. (2022). Durability of Eco-Friendly Blended Cements Incorporating Ceramic Waste from Different Sources. J. Sustain. Cement-Based Mater..

[19] Mohammadhosseini H., Lim N.H.A.S., Tahir M.M., Alyousef R., Samadi M., Alabduljabbar H. (2020). Effects of Waste Ceramic as Cement and Fine Aggregate on Durability Performance of Sustainable Mortar. Arab. J. Sci. Eng..

[20] Ikotun J.O., Adedeji P.O., Babafemi A.J. (2025). A Comprehensive Review on the Performance of Low-Carbon Ceramic Waste Powder as Cement Replacement Material in Concrete. Appl. Sci..

[21] Scrivener K., Martirena F., Bishnoi S., Maity S. (2018). Calcined Clay Limestone Cements (LC3). Cem. Concr. Res..

[22] Mañosa J., Calderón A., Salgado-Pizarro R., Maldonado-Alameda A., Chimenos J.M. (2024). Research Evolution of Limestone Calcined Clay Cement (LC3), a Promising Low-Carbon Binder—A Comprehensive Overview. Heliyon.

[23] Ijaz N., Ye W.-M., ur Rehman Z., Ijaz Z., Junaid M.F. (2024). Global Insights into Micro-Macro Mechanisms and Environmental Implications of Limestone Calcined Clay Cement (LC3) for Sustainable Construction Applications. Sci. Total Environ..

[24] Sharma M., Bishnoi S., Martirena F., Scrivener K. (2021). Limestone Calcined Clay Cement and Concrete: A State-of-the-Art Review. Cem. Concr. Res..

[25] Mohit M., Haftbaradaran H., Tajmir Riahi H. (2023). Investigating the Ternary Cement Containing Portland Cement, Ceramic Waste Powder, and Limestone. Constr. Build. Mater..

[26] De Matos P.R., Doerner G., da Silva Nazário S., da Silva Andrade Neto J., Longhi M., Folgueras M., Rodríguez E.D. (2025). Limestone Calcined Clay Cements (LC3) Produced with Iron Ore Tailings and Ceramic Waste: Hydration, Mechanical Performance and Rheology. Constr. Build. Mater..

[27] Marangu J.M. (2020). Effects of Sulfuric Acid Attack on Hydrated Calcined Clay–Limestone Cement Mortars. J. Sustain. Cement-Based Mater..

[28] Tokareva A., Kaassamani S., Waldmann D. (2024). Using Ceramic Demolition Wastes for CO_2_-Reduced Cement Production. Constr. Build. Mater..

[29] (2016). Methods of Testing Cement—Part 1: Determination of Strength.

[30] Raverdy M., Brivot F., Paillere A.M., Dron R. Appréciation de l’activité pouzzolanique des constituants secondaires. Proceedings of the 7th International Congress on the Chemistry of Cement.

[31] (2012). Addition for Concrete—Metakaolin—Specifications and Conformity Criteria.

[32] (2022). Standard Specification for Coal Fly Ash and Raw or Calcined Natural Pozzolan for Use in Concrete.

[33] Indhumathi S., Kumar S.P., Pichumani M. (2022). Reconnoitring Principles and Practice of Modified Andreasen and Andersen Particle Packing Theory to Augment Engineered Cementitious Composite. Constr. Build. Mater..

[34] Yin T., Liu K., Fan D., Yu R. (2023). Derivation and Verification of Multilevel Particle Packing Model for Ultra-High Performance Concrete (UHPC): Modelling and Experiments. Cem. Concr. Compos..

[35] Liu K., Yin T., Fan D., Wang J., Yu R. (2022). Multiple Effects of Particle Size Distribution Modulus (q) and Maximum Aggregate Size (Dmax) on the Characteristics of Ultra-High Performance Concrete (UHPC): Experiments and Modeling. Cem. Concr. Compos..

[36] Brouwers H.J.H., Radix H.J. (2005). Self-Compacting Concrete: Theoretical and Experimental Study. Cem. Concr. Res..

[37] (2009). Cement—Test Methods—Determination of Strength.

[38] (2005). Admixtures for Concrete, Mortar and Grout—Test Methods—Part 5: Determination of Capillary Absorption.

[39] (2002). Products and Systems for the Protection and Repair of Concrete Structures—Test Methods—Part 4: Determination of Shrinkage and Expansion.

[40] Tokareva A., Waldmann D. (2025). Durability of Cement Mortars Containing Fine Demolition Wastes as Supplementary Cementitious Materials. Constr. Build. Mater..

[41] Skibsted J., Snellings R. (2019). Reactivity of Supplementary Cementitious Materials (SCMs) in Cement Blends. Cem. Concr. Res..

[42] Li L., Joseph P., Zhang X., Zhang L. (2024). A Study of Some Relevant Properties of Concrete Incorporating Waste Ceramic Powder as a Cement Replacement Agent. J. Build. Eng..

[43] Pacheco-Torgal F., Jalali S. (2010). Reusing ceramic wastes in concrete. Constr. Build. Mater..

[44] Nasir M., Alimi W.O., Oladapo E.A., Imran M., Kazmi Z.A. (2023). Behavior of Drying and Plastic Shrinkage of Portland Cement Concrete Prepared and Cured under Harsh Field. Dev. Built Environ..

[45] Zhou C., Zhang X., Qiao J., Feng J., Zeng Q. (2025). The Deformation of CSH Gels and Its Link with Dynamic Length Change of Cement Pastes upon Drying and Resaturation. Cem. Concr. Res..

[46] McDonald P.J., Istok O., Janota M., Gajewicz-Jaromin A.M., Faux D.A. (2020). Sorption, Anomalous Water Transport and Dynamic Porosity in Cement Paste: A Spatially Localised 1H NMR Relaxation Study and a Proposed Mechanism. Cem. Concr. Res..

[47] Alderete N.M., Villagrán Zaccardi Y.A., De Belie N. (2019). Physical Evidence of Swelling as the Cause of Anomalous Capillary Water Uptake by Cementitious Materials. Cem. Concr. Res..

[48] Miao Y., Yu W., Jin L., Wang L., Lin J., Li Y., Lu Z., Jiang J. (2024). Effect of Shrinkage-Induced Initial Damage on the Frost Resistance of Concrete in Cold Regions. Eng. Fract. Mech..

[49] Rhardane A., Sleiman S.A.H., Alam S.Y., Grondin F. (2021). A Quantitative Assessment of the Parameters Involved in the Freeze–Thaw Damage of Cement-Based Materials through Numerical Modelling. Constr. Build. Mater..

[50] Poon C.-S., Azhar S., Anson M., Wong Y.-L. (2001). Comparison of the Strength and Durability Performance of Normal- and High-Strength Pozzolanic Concretes at Elevated Temperatures. Cem. Concr. Res..

[51] Heikal M. (2000). Effect of Temperature on the Physico-Mechanical and Mineralogical Properties of Homra Pozzolanic Cement Pastes. Cem. Concr. Res..

[52] Seleem H.E.H., Rashad A.M., Elsokary T. (2011). Effect of Elevated Temperature on Physico-Mechanical Properties of Blended Cement Concrete. Constr. Build. Mater..

[53] Chong R., Zhang W., Yin B., Liew K.M. (2025). Evaluating Next-Gen Sustainable Cementitious Materials: Unleashing the Potential of LC3-Based Composites under High-Temperature Environments. J. Clean. Prod..

[54] Lin R.-S., Han Y., Wang X.-Y. (2021). Macro–Meso–Micro Experimental Studies of Calcined Clay Limestone Cement (LC3) Paste Subjected to Elevated Temperature. Cem. Concr. Compos..

[55] Keyser H., Swenson E.G. (1968). Scaling of Concrete by Frost Action. Performance of Concrete: Resistance of Concrete to Sulphate and Other Environmental Conditions. A Symposium in Honour of Thorbergur Thorvaldson.

[56] Jedidi M. (2024). Effect of Temperature Rise Caused by Fire on the Physical and Mechanical Properties of Concrete. Insight-Civ. Eng..

[57] Mindess S., Hewlett P.C., Liska M. (2019). Resistance of Concrete to Destructive Agencies. Lea’s Chemistry of Cement and Concrete.

[58] Liew J.Y.R., Xiong M.-X., Lai B.-L. (2021). Design of Steel-Concrete Composite Structures Using High Strength Materials.

[59] Cao R., Yang J., Li G., Liu F., Niu M., Wang W. (2022). Resistance of the Composite Cementitious System of Ordinary Portland/Calcium Sulfoaluminate Cement to Sulfuric Acid Attack. Constr. Build. Mater..

[60] Khan H.A., Castel A., Khan M.S.H., Mahmood A.H. (2019). Durability of Calcium Aluminate and Sulphate Resistant Portland Cement Based Mortars in Aggressive Sewer Environment and Sulphuric Acid. Cem. Concr. Res..

[61] Rozière E., Loukili A., El Hachem R., Grondin F. (2009). Durability of Concrete Exposed to Leaching and External Sulphate Attacks. Cem. Concr. Res..

[62] Gu L., Bennett T., Visintin P. (2019). Sulphuric Acid Exposure of Conventional Concrete and Alkali-Activated Concrete: Assessment of Test Methodologies. Constr. Build. Mater..

[63] Saputra A.H., Shohibi M., Kubouchi M. (2015). Effect of Fly Ash Fortification in the Manufacture Process of Making Concrete Towards Characteristics of Concrete in Sulfuric Acid Solution. Makara J. Technol..

[64] Sinkhonde D., Onchiri R.O., Oyawa W.O., Mwero J.N. (2022). Durability and Water Absorption Behaviour of Rubberised Concrete Incorporating Burnt Clay Brick Powder. Clean. Mater..

